# Mechanisms and clinical advancements of cell-based immunotherapies in non-small cell lung cancer: an integrated perspective

**DOI:** 10.3389/fimmu.2025.1633100

**Published:** 2025-08-19

**Authors:** Lixian Yang, Yaoyao Gong

**Affiliations:** ^1^ Department of Second Clinical College, Cheeloo College of Medicine, Shandong University, Jinan, Shandong, China; ^2^ Department of Cardiology, The Second Hospital, Cheeloo College of Medicine, Shandong University, Jinan, Shandong, China

**Keywords:** non-small cell lung cancer, clinical trials, cell therapy, lymphocyteactivated killer cell therapy, tumor-infiltrating lymphocyte cell therapy, dendritic cellcytokine-induced killer cell therapy, chimeric antigen receptor T cell therapy

## Abstract

Non-small cell lung cancer (NSCLC) remains a leading cause of cancer-related mortality worldwide, with only modest improvements in survival despite advances in conventional therapies. Cell-based immunotherapy, which utilizes ex vivo expanded or genetically modified immune cells, has emerged as a promising therapeutic alternative. Approaches such as natural killer (NK) cells, tumor-infiltrating lymphocytes (TILs), dendritic cell (DC)-based vaccines, cytokine-induced killer (CIK) cells, and chimeric antigen receptor T (CAR-T) cells have shown encouraging potential in preclinical and early clinical studies. However, their clinical efficacy in NSCLC is significantly constrained by multiple factors, including the immunosuppressive tumor microenvironment (TME), intratumoral antigenic heterogeneity, and limited persistence and expansion of adoptively transferred cells. To address these barriers, advances in cellular engineering, rational combinatorial regimens, and refined patient selection strategies are actively being explored. This review provides a critical overview of the current landscape of cell-based therapies in NSCLC, focusing on recent breakthroughs, persistent limitations, and evolving strategies to enhance therapeutic outcomes. By contextualizing these developments, we aim to clarify the translational potential of cellular immunotherapy and its role in redefining the treatment paradigm for NSCLC.

## 1 Introduction

Non-small cell lung cancer (NSCLC) accounts for approximately 85% of all lung cancer cases and remains the most prevalent and lethal malignancy worldwide, with an estimated 2 million new cases and 1.76 million deaths annually (1, 2). According to the National Comprehensive Cancer Network (NCCN) guidelines, surgical resection with lymph node dissection is the primary treatment for resectable NSCLC. In cases where no residual tumor is detected postoperatively, adjuvant therapies such as Atezolizumab or Osimertinib may be considered (3). However, for patients ineligible for surgery, concurrent radiotherapy and chemotherapy are recommended, with Durvalumab used as the standard adjuvant treatment (3). Despite these therapeutic advances, the 5-year overall survival (OS) rate for NSCLC remains below 5% in advanced stages, primarily due to micrometastases that are already present at the time of diagnosis and the inability of localized therapies to eradicate all malignant cells (4). Therefore, there is an urgent need to develop more effective and targeted treatment strategies to overcome these limitations.

In recent years, cell-based immunotherapy has emerged as a promising modality for NSCLC treatment. Originally explored in hematologic malignancies, adoptive cell therapy (ACT) has since been investigated in solid tumors, including NSCLC (5). This approach utilizes ex vivo expanded or genetically modified immune cells to enhance tumor-targeted immune responses. Among the various strategies, natural killer (NK) cells, tumor-infiltrating lymphocytes (TILs), dendritic cell (DC)-based vaccines, cytokine-induced killer (CIK) cells, and chimeric antigen receptor T (CAR-T) cells have shown potential therapeutic benefits in both preclinical and clinical studies (5–9). NK cells exhibit innate cytotoxicity against tumor cells through major histocompatibility complex (MHC)-independent mechanisms, making them a key component of cell-based immunotherapy (10). Building on NK cell-based strategies, lymphokine-activated killer cell (LAK) therapy was developed to enhance cytotoxic activity against solid tumors (11, 12). This approach, pioneered by Rosenberg et al., utilizes interleukin-2 (IL-2)-activated peripheral blood lymphocytes to amplify the tumor-killing capacity of NK and T cells (11, 12). Further advancing this concept, TIL-based therapy demonstrated that large-scale expansion of TILs could improve antitumor efficacy in metastatic cancers (13). Likewise, DC-based immunotherapy has gained attention due to the central role of dendritic cells as antigen-presenting cells. While early attempts using autologous DCs showed limited success, DC-CIK combination therapy significantly enhanced tumor-specific cytotoxicity and cytokine secretion, particularly in hematological malignancies (14, 15). Meanwhile, CAR-T cell therapy has transformed cancer immunotherapy. With continuous advancements across five generations, CAR-T technology has improved specificity and efficacy, extending its application beyond hematologic malignancies (16).

Despite these advances, significant challenges remain in translating cell therapies into effective NSCLC treatment. The immunosuppressive tumor microenvironment (TME), tumor antigen heterogeneity, and limited persistence of infused cells have hindered the efficacy of these therapies in solid tumors. Additionally, while clinical trials have demonstrated encouraging results, the therapeutic response in NSCLC remains less robust than in hematologic cancers, highlighting the need for further optimization of cell engineering strategies, combination regimens, and patient selection. Given the growing interest in cell-based therapies for NSCLC, this review aims to provide a comprehensive analysis of their underlying mechanisms, summarize key clinical trials, and discuss ongoing challenges and future directions. By critically evaluating current evidence, this review seeks to offer insights into the clinical application of cell therapy and its potential to complement or overcome the limitations of conventional treatments.

## 2 LAK cell therapy

### 2.1 Mechanism of LAK cell therapy

**Figure 1:**
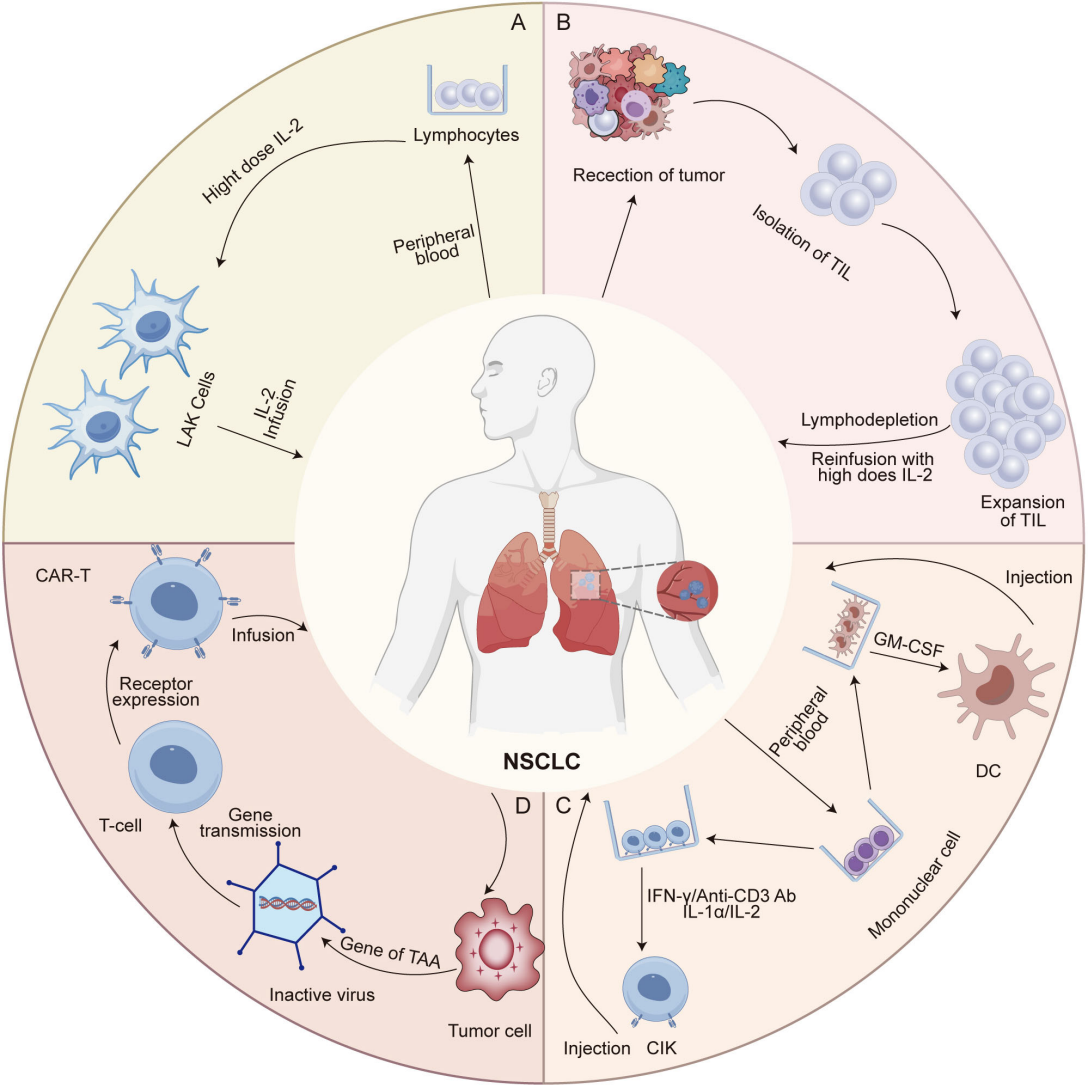
Preparation and mechanisms of action of major adoptive cell therapies in NSCLC. **(A)** Lymphokine-activated killer (LAK) cells are generated by culturing peripheral blood lymphocytes (PBLs) with high-dose interleukin-2 (IL-2), leading to the expansion of non-specific cytotoxic effector cells. LAKs exert anti-tumor activity through direct cytolysis of tumor cells, although their clinical efficacy is limited due to lack of tumor specificity. **(B)** Tumor-infiltrating lymphocytes (TILs) are isolated from resected tumor tissues and expanded ex vivo using IL-2. These cells retain tumor specificity and recognize tumor antigens via native T cell receptors (TCRs), enabling antigen-dependent cytotoxicity upon reinfusion. **(C)** Dendritic cell-cytokine-induced killer (DC-CIK) cells are generated by co-culturing autologous dendritic cells with cytokine-induced killer cells, which are expanded from PBLs using IFN-γ, anti-CD3 antibodies, and IL-2. The DCs present tumor-associated antigens to prime the CIK cells, enhancing their MHC-unrestricted cytotoxicity. **(D)** T cells derived from the patient or a donor are collected and genetically modified *in vitro* to express chimeric antigen receptors (CARs). These engineered T cells are then expanded ex vivo and reinfused into the patient, where they specifically recognize and eliminate tumor cells through CAR-mediated antigen targeting.

Lymphokine-activated killer (LAK) cell therapy involves the ex vivo activation of peripheral blood lymphocytes (PBLs) with high-dose interleukin-2 (IL-2), generating a heterogeneous population of NK cells and non-MHC-restricted cytotoxic T lymphocytes (CTLs) (**Figure 1A**) (17, 18). These cells exert antitumor effects by recognizing stress-induced ligands (e.g., MICA/B, ULBP) and inducing apoptosis through perforin/granzyme and Fas/FasL pathways (17, 18). IL-2 not only enhances cytotoxicity but also promotes cytokine release, including interferon-gamma (IFN-γ) and tumor necrosis factor-alpha (TNF-α), contributing to macrophage repolarization and immune modulation (19). However, reliance on high-dose IL-2 frequently results in off-target effects and nonspecific immune activation, thereby limiting its clinical applicability. For instance, studies have indicated that LAK-induced cytotoxicity may extend to nonmalignant tissues, including endothelial cells and normal lymphocytes (20). This off-target activity is primarily attributed to the low-level expression of stress ligands in healthy tissues and IL-2-induced bystander activation of autoreactive lymphocytes. These mechanistic limitations provide context for the modest efficacy and safety concerns observed in clinical trials.

### 2.2 Clinical trials of LAK cell therapy

A comprehensive evaluation of clinical trials (**Table 1**) reveals that the therapeutic efficacy of LAK cell therapy is principally determined by three mechanistic factors: IL-2 dose optimization, cellular delivery strategies, and integration with conventional treatments. The IL-2/LAK axis exhibits a narrow therapeutic window. For instance, in a phase II trial, Bernstein et al. reported that high-dose IL-2 administration (6×10^6^ U/kg/day) resulted in dose-limiting pulmonary toxicity in nine of eleven patients with advanced NSCLC, yielding only an 18% partial response rate (21). Subsequent trials with pharmacokinetic modulation demonstrated that reducing the IL-2 dose tenfold (6×10^5^ U/kg) and applying intermittent LAK reinfusion could enhance efficacy while minimizing toxicity, achieving a 40% objective response rate (22). This dose-dependent balance reflects the dual role of IL-2. While high doses enhance LAK cell expansion and cytotoxicity, they also induce systemic toxicities such as vascular leak syndrome, largely due to endothelial activation and nonspecific cytokine release. In contrast, low doses may fail to sustain sufficient immune activation. Thus, optimizing IL-2 dosing is critical to maximize antitumor efficacy while minimizing adverse effects. Cell delivery routes also significantly impact tumor infiltration. A pivotal randomized controlled trial (RCT) by Watanabe et al. showed that bronchial artery infusion, following chemoimmunotherapy, led to improved survival compared to systemic administration, likely due to enhanced regional accumulation (23). This was likely due to improved regional accumulation of cytotoxic lymphocytes. By contrast, a phase II postoperative study by Yano et al. found no survival benefit with subcutaneous IL-2 plus delayed LAK administration, suggesting that timing and anatomical targeting are crucial (24).

**Table 1:** Summary of key clinical trials on LAK cell therapy.

Author (Year)	Study design	Patient population	Treatment regimen	Key outcomes	Challenges and indication
Bernstein et al. (1991) (21)	Phase II	Advanced NSCLC (n=11)	High-dose IL-2 (6×10^6^ U/kg/day) + continuous LAK cells	-ORR: 18% - 9/11 experienced dose-limiting pulmonary toxicity	High-dose IL-2 has a narrow therapeutic window. Dose optimization is necessary.
Sparano et al. (1994) (22)	Phase II	Advanced NSCLC (n=6)	Low-dose IL-2 (6×10^5^ U/kg) + intermittent LAK infusions	- ORR: 40% - Markedly improved tolerability	Tenfold IL-2 dose reduction maintained efficacy and reduced risk of vascular leak syndrome.
Watanabe et al. (1989) (23)	RCT	Lung cancer (n=305)	Bronchial arterial + chemo/immunotherapy vs. systemic LAK infusion + chemo/immunotherapy	- Superior survival in arterial infusion group	Regional delivery improved TME localization of effector cells, enhancing therapeutic effect.
Yano et al. (1999) (24)	Phase II	NSCLC postoperative patients (n=40)	Subcutaneous IL-2 + delayed LAK infusion vs. standard therapy	- No significant survival benefit	Delayed timing and subcutaneous administration limited effective cell-tumor interaction.
Kimura et al. (1995) (102)	Phase II	NSCLC with non-curative resection	Postoperative IL-2/LAK vs. observation	- 7-year OS: 39.1% (treatment) vs. 12.7% (control)	LAK therapy eliminated MRD and early postoperative IL-2 expanded endogenous TILs.
Kimura et al. (1997) (26)	Phase III	Primary lung carcinoma patients postsurgically (n=174)	Chemo/radiotherapy + IL-2/LAK vs. standard therapy	- 5-year OS: 54.4% vs. 33.4% - 9-year OS: 52.0% vs. 24.2%	Multimodal synergy: chemotherapy-induced immunogenic cell death; LAK cleared residuals; IL-2 enhanced systemic immunity.

NSCLC, Non-Small Cell Lung Cancer; IL-2, Interleukin-2; LAK, Lymphokine-Activated Killer; ORR, Objective Response Rate; RCT, Randomized Controlled Trial; TME, Tumor Microenvironment; OS, Overall Survival; MRD, Minimal Residual Disease; TIL, Tumor-Infiltrating Lymphocyte.

Combining LAK therapy with other treatments has shown additional benefit. In a phase II study, Kimura et al. found that adjuvant IL-2/LAK post-non-curative surgery tripled the 7-year survival rate (39.1% vs. 12.7%, *p*<0.01) (25). A follow-up phase III trial reported 5-year and 9-year survival rates of 54.4% and 52.0%, respectively, with IL-2/LAK plus chemotherapy or radiotherapy, compared to 33.4% and 24.2% in controls (26). These effects are likely due to: (1) elimination of residual disease by infused LAK cells, (2) IL-2-mediated expansion of endogenous TILs, and (3) systemic immune priming through immunogenic cell death from chemotherapy. These findings support multimodal integration to enhance LAK therapy outcomes.

### 2.3 Barrier and promise of LAK cell therapy

Despite mechanistic rationale, clinical results for LAK therapy in NSCLC remain mixed. Though often co-administered with IL-2, its efficacy is undermined by the TME, particularly transforming growth factor-beta (TGF-β), programmed death-ligand 1 (PD-L1) expression, and poor tumor-homing ability. Intrapleural infusion has improved control of malignant pleural effusion, yet survival gains are limited. Moreover, IL-2-related toxicities, including vascular leak syndrome, and the short persistence of infused cells (typically under 1 week) restrict durable responses. To improve outcomes, strategies include IL-2 dose refinement, precise delivery, and combinations with cytoreductive therapies. Emerging approaches, such as cytokine engineering and tumor-directed lymphocyte modification, may address these issues partially. Nonetheless, due to non-specific cytotoxicity and limited durability, LAK therapy is now largely being replaced by antigen-specific, next-generation cell therapies with better precision and long-term efficacy.

## 3 TIL cell therapy

### 3.1 Mechanism of TIL cell therapy

Tumor-infiltrating lymphocyte (TIL) therapy is a personalized adoptive cell therapy that utilizes autologous T cells isolated from resected tumor tissues (**Figure 1B**) (27, 28). These TILs exhibit tumor specificity by recognizing patient-specific neoantigens presented on MHC molecules, making them especially effective in heterogeneous solid tumors (27, 28). Following surgical excision, TILs are expanded ex vivo using high-dose recombinant IL-2 and reinfused into the patient after a lymphodepleting regimen, typically cyclophosphamide and fludarabine (27). This preparative step reduces immunosuppressive cells and enhances cytokine availability, thereby improving TIL persistence and engraftment (27, 29). While conventional 7-day lymphodepletion regimens are associated with severe toxicities, emerging studies using shorter protocols (3-5 days) have shown comparable efficacy with improved safety profiles (30–32). After infusion, IL-2 promotes TIL survival via signal transducer and activator of transcription-5 (STAT5) signaling, while chemokine receptors like C-X-C chemokine receptor 3 (CXCR3) and C-C chemokine receptor 5 (CCR5) guide TIL migration to tumor sites (33). Upon arrival, TILs exert cytotoxic effects through perforin/granzyme pathways and Fas/FasL interactions. In addition, TIL-derived cytokines such as IFN-γ and TNF-α contribute to the remodeling of the TME, including macrophage repolarization and increased antigen presentation (34).

A key advantage of TIL therapy lies in its use of autologous lymphocytes isolated directly from tumor tissue, preserving a polyclonal T-cell repertoire capable of recognizing a diverse array of tumor neoantigens (28). This intrinsic diversity allows TILs to effectively address tumor heterogeneity and antigen escape, barriers that often undermine the efficacy of CAR-T or NK-based therapies in solid tumors. Unlike CAR-T cells, which are engineered to target a single epitope, TILs exploit the full spectrum of endogenous immune recognition without requiring genetic modification (35). They have shown durable clinical responses, particularly in tumors resistant to immune checkpoint inhibitors (ICIs), such as metastatic melanoma and cervical cancer (35). Emerging data in NSCLC suggest similar potential, especially in heavily pretreated or ICIs-refractory patients. Despite manufacturing and scalability challenges, these mechanistic strengths support the continued clinical development of TIL therapy in solid tumors (29, 30). The following section reviews key clinical studies that highlight its promise in NSCLC.

### 3.2 Clinical trials of TIL cell therapy

As shown in **Table 2**, the foundational role of IL-2 in supporting adoptively transferred TILs was demonstrated in early NSCLC studies, where TILs could be successfully expanded from minimally invasive tumor biopsies (36). Continuous IL-2 infusion promoted *in vivo* T-cell proliferation and persistence, underscoring the importance of cytokine support for TIL viability. However, these trials also revealed IL-2’s dual role: while enhancing effector function, it can also expand immunosuppressive regulatory T cells (Tregs), warranting optimized cytokine protocols (36). Subsequent RCTs, including one involving 131 patients with resectable NSCLC, further advanced the field by evaluating postoperative TIL therapy in combination with subcutaneous IL-2 administration (37). The stage-specific benefit, notably in stage IIIB patients (*p*<0.05), was attributed to TIL-mediated clearance of micrometastases (37). In contrast, earlier-stage patients derived limited benefit, suggesting that tumor antigen load and immune infiltration influence TIL efficacy (37). More recent trials have combined TILs with ICIs. A phase I study (NCT03215810) evaluated TILs plus nivolumab in PD-1-refractory NSCLC (38). Among 20 patients, the objective response rate (ORR) was 23.1% (3/13 evaluable) and 84.6% showed tumor burden reduction, with acceptable toxicity (≤17% grade≥3) (38). These findings indicate that PD-1 blockade may reverse exhaustion in reinfused TILs, restoring their cytolytic function and enhancing therapeutic outcomes. Moreover, the autologous TIL product Lifileucel (LN-145) was evaluated in a phase II multicenter trial (NCT03645928), enrolling 28 metastatic NSCLC patients post-ICI therapy (39). The ORR was 21.4% (6/28), including responses in PD-L1-negative, low tumor mutational burden (TMB), and STK11-mutated tumors, indicating potential benefit in difficult-to-treat subsets (39). The trial has since expanded to include multiple cohorts evaluating Lifileucel alone or with ICIs (e.g., nivolumab, pembrolizumab) in advanced solid tumors, including NSCLC. Although OS data are pending, interim results support further exploration in ICI-refractory cases. These findings underscore the retained functional plasticity of autologous TILs, even in checkpoint inhibitor-resistant disease settings.

**Table 2:** Summary of key clinical trials on TIL cell therapy.

Author (Year)	Study design	Patient population	Treatment regimen	Key outcomes	Challenges and indication
Kradin et al. (1989) (36)	Phase I	NSCLC patients (n=8)	TIL + continuous IL-2 infusion	-Limited toxicity but subtle therapeutic effect	IL-2 sustains TILs; dual role: activates effectors and expands Tregs
Ratto et al. (1996) (37)	Phase II	Postoperative NSCLC patients (n=131)	TIL + subcutaneous IL-2 vs. standard chemoradiotherapy	-Reduced local recurrence in stage IIIB; -Limited benefit in early-stage patients	Efficacy linked to tumor antigen load; lack of predictive biomarkers
Creelan et al. (2021) (38) NCT03215810	Phase I	Anti-PD-1–resistant NSCLC patients (n=20)	TIL + Nivolumab	-ORR 23.1% (3/13); -Tumor burden reduced in 84.6%; Grade ≥3 toxicity ≤17%	Small sample; efficacy in PD-L1-negative and low-TMB subgroups unproven
Schoenfeld et al. (2024) (39) NCT03645928	Phase II	metastatic NSCLC (PD-L1^–^, low TMB, STK11-mutant) (n=28)	Lifileucel (LN-145)	-ORR 21.4% (6/28); -Included immunotherapy-resistant subtypes	Long-term survival pending; 4–6 week manufacturing limits clinical use
DNT Cell Study	Preclinical		DNT Cells + PD-1 blockade	-Strong cytotoxicity against lung cancer cells; -Enhanced effect with PD-1 blockade	Not yet tested clinically; needs validation for safety and tumor homing
NCT02133196	Phase II	Patients with NSCLC whose tumors can be safely resected	Young TILs + chemotherapy	-Trial ongoing, which seeks to optimize expansion and combination strategies	Results pending; TIL heterogeneity and cost remain key challenges

NSCLC, Non-Small Cell Lung Cancer; TIL, Tumor-Infiltrating Lymphocyte; IL-2, Interleukin-2; ORR, Objective Response Rate; PD-1, Programmed Cell Death Protein 1; PD-L1, Programmed Death-Ligand 1; TMB, Tumor Mutational Burden; STK11, Serine/Threonine Kinase 11; DNT, Double Negative T (cells); LN-145, Lifileucel.

Beyond conventional TIL populations, emerging T-cell subsets such as double-negative T (DNT) cells (CD3^+^CD4^-^CD8^-^) have demonstrated potent cytotoxic activity against lung cancer in both *in vitro* and xenograft models. When combined with PD-1 blockade, DNT cells exhibit enhanced efficacy, suggesting a promising avenue for future ACT strategies (40). In parallel, ongoing phase II trials (NCT02133196) are investigating the therapeutic potential of young TILs in combination with agents such as Aldesleukin, Fludarabine, and Cyclophosphamide, aiming to refine treatment protocols and broaden the clinical applicability of TIL-based immunotherapies in NSCLC and other solid tumors.

### 3.3 Barrier and promise of TIL cell therapy

While TIL therapy offers durable responses and antigen diversity, barriers such as product heterogeneity, manufacturing variability, and TME-driven suppression must be addressed for broader clinical translation. Despite its strengths, several challenges limit the widespread adoption of TIL therapy. One major issue is the absence of validated biomarkers for predicting manufacturing success or clinical efficacy (41). Although current protocols achieve a manufacturing success rate exceeding 90%, occasional failures, often associated with toxic lymphodepletion regimens, highlight the urgent need for reliable in-process control markers (41). Metrics such as expansion indices or real-time cytotoxicity assays are being explored to evaluate TIL product viability prior to infusion (34). While biomarkers commonly used in immune checkpoint blockade, such as TMB or CD8^+^CD27^+^ T-cell frequency, have been investigated, their applicability in the context of TIL therapy remains limited (42, 43). In contrast, single-cell functional assays are emerging as promising tools for predicting product potency and therapeutic outcomes (34). Another critical barrier lies in the intrinsic heterogeneity of the TIL population. Despite the enrichment of tumor-reactive clones during expansion, the final product is frequently dominated by non-specific or bystander T-cell populations, which can dilute overall antitumor activity (44, 45). This underscores the need for refined strategies such as clonal selection or selective expansion protocols to enhance the specificity and cytotoxic potential of the TIL product. Furthermore, the absence of a clear dose-response relationship complicates efforts to optimize treatment efficacy. The immunosuppressive TME, characterized by T-cell terminal differentiation, metabolic exhaustion, and inhibitory signaling pathways, also significantly impairs TIL persistence and function post-infusion.

To overcome these barriers, combination strategies have gained increasing attention. In particular, the co-administration of PD-1/PD-L1 inhibitors has shown the potential to reverse T-cell exhaustion and restore cytolytic activity, thereby improving therapeutic outcomes (38). In addition, novel approaches such as metabolic reprogramming, including antioxidant supplementation to mitigate oxidative stress, and the engineering of TIL subsets with favorable phenotypes including BTLA-low effector T cells exhibiting reduced inhibitory signaling are being developed to enhance efficacy and durability (46). Despite existing challenges in standardization, scalability, and toxicity management, ongoing advances in manufacturing technology, biomarker-guided patient selection, and combinatorial regimens are paving the way for TIL therapy to emerge as a viable and promising treatment option, particularly for patients with refractory NSCLC and other solid tumors.

## 4 DC/CIK cell therapy

### 4.1 Mechanism of DC/CIK cell therapy

Dendritic cell (DC) and cytokine-induced killer (CIK) cell immunotherapy represents a promising form of adoptive cell therapy, involving the ex vivo expansion and activation of autologous immune cells (**Figure 1C**) (47). This approach integrates the potent antigen-presenting capabilities of DCs with the cytotoxic potential of CIKs (47). DCs capture tumor-associated antigens (TAAs) via phagocytosis or receptor-mediated uptake, process them into antigenic peptides, and present them in the context of MHC-I/II molecules, thereby initiating antigen-specific T-cell responses within lymphoid tissues (48). In addition, DCs express key co-stimulatory molecules, including CD80/CD86 and CD40, and secrete immunostimulatory cytokines such as IL-12 and IL-15, which drive Th1 polarization and enhance CD8^+^ T-cell cytotoxicity (49). CIK cells, primarily consisting of CD3^+^CD56^+^ T-cell subsets generated by stimulation with IFN-γ, IL-2, and anti-CD3 monoclonal antibodies, mediate tumor cell killing through both the perforin/granzyme B pathway and death receptor-mediated signaling such as Fas/FasL (50). These cells also secrete IFN-γ and TNF-α, which inhibit tumor angiogenesis and induce M1 macrophage polarization (51). Furthermore, CIK cells exert immunomodulatory effects by targeting immunosuppressive cells such as Tregs and myeloid-derived suppressor cells (MDSCs), thereby partially reversing the immunosuppressive TME (51). The interplay between DCs and CIK cells forms a positive feedback loop: DCs activate and expand CIK-like effectors, while cytokines from CIK cells promote DC maturation and antigen presentation, collectively enhancing antitumor immunity. These synergistic mechanisms have provided a rationale for clinical trials assessing DC/CIK therapy in NSCLC, particularly in combination with other modalities.

### 4.2 Clinical trials of DC/CIK cell therapy

Clinical studies have shown that DC/CIK therapy can reshape systemic immune profiles and improve therapeutic efficacy in patients with advanced NSCLC (**Table 3**). One randomized trial reported post-treatment increases in circulating CD3^+^, CD4^+^, CD8^+^ T cells and NK cells in patients receiving DC/CIK therapy, correlating with prolonged progression-free survival (3.20 vs. 2.56 months; *p*<0.05) (52). However, some patients exhibited a persistent Th2-biased immune profile, suggesting incomplete Th1 polarization, which may compromise durable antitumor responses (53). In a paired cohort study involving stage III-IV patients, chemotherapy combined with DC/CIK therapy significantly improved 1- and 2-year overall survival (57.2% and 27.0% vs. 37.3% and 10.1%; *p*<0.05), with no significant difference between adenocarcinoma and squamous cell carcinoma, although no stratification by tumor stage was provided (54). Another randomized trial focusing on postoperative patients with pathological stages IB-IV found that DC/CIK-based adjuvant immunotherapy led to 2- and 5-year overall survival rates of 93.4% and 81.4%, respectively, markedly higher than 66.0% and 48.3% in the chemotherapy-alone group (55). These benefits were achieved with only mild, transient adverse events. Importantly, this trial included patients with high-risk features such as pleural dissemination or micrometastasis, supporting the potential benefit of DC/CIK therapy across a range of early to advanced stages, though detailed subgroup survival analysis was lacking (55).

**Table 3:** Summary of key clinical trials on DC/CIK cell therapy.

Author (Year)	Study design	Patient population	Treatment regimen	Key outcomes	Challenges and indication
Shi et al. (2012) (52)	RCT	Stage IIIB and IV NSCLC patients stabilized following platinum treatment (n=60)	DC/CIK vs. control	-Significant increase in NK cells and T cell subsets (CD3^+^, CD4^+^, CD8^+^); -PFS prolonged (3.20 vs. 2.56 months, p<0.05)	Persistent Th2 microenvironment; incomplete Th1 polarization
Yang et al. (2013) (54)	RCT	Advanced NSCLC (n=61)	DC/CIK + platinum-based chemotherapy vs. chemotherapy alone	-Increased CD3^+^/CD56^+^ cell ratio; -2-year OS significantly improved (27.0% vs. 10.1%, p<0.05)	No stratification by histological subtype; limited long-term survival data
Kimura et al. (2015) (55)	Phase III RCT	Postsurgical NSCLC (n=103)	DC/CIK + chemotherapy vs. chemotherapy alone	-2-year OS: 93.4% vs. 66.0%; -5-year OS: 81.4% vs. 48.3% (p<0.05)	Only mild AEs reported (fever, chills); lack of biomarker-based patient selection
Zhong et al. (2014) (56)	RCT	Stage IIIB and IV NSCLC patients (n=60)	DC/CIK; Stratified by treatment cycles (≤2 vs. >2)	-Longer median TTP in >2-cycle group (7.3 vs. 6.2 months p<0.05); -3-year OS improved (23.3% vs. 6.7%, p<0.05)	Skin toxicity and non-infectious fever require management; lacks prospective validation
Zhu et al. (2015) (57)	RCT	Stage IIIB NSCLC (n=63)	DC/CIK + docetaxel-cisplatin and conformal radiotherapy vs. chemoradiotherapy	-ORR 83.3% vs. 54.5% (p<0.05); -12-month survival rate increased (63.3% vs. 42.4%)	Long-term survival not evaluated; no significant increase in treatment toxicity
Zhou et al. (2022) (58) NCT03987867	Phase I	stage IIIB/IIIC/IV NSCLC (n=34)	CIK + chemotherapy + Sintilimab	-ORR 82.4%, DCR 100%; -Median PFS 19.3 months	94.1% experienced treatment-related AEs; one grade 5 immune pneumonia death; further safety validation needed

NSCLC, Non-Small Cell Lung Cancer; DC, Dendritic Cell; CIK, Cytokine-Induced Killer (cell); RCT, Randomized Controlled Trial; OS, Overall Survival; PFS, Progression-Free Survival; TTP, Time to Progression; ORR, Objective Response Rate; DCR, Disease Control Rate; AEs, Adverse Events; NK, Natural Killer (cell).

Therapy frequency also impacts clinical benefit. In a cohort of stage IIIB-IV patients, those receiving more than two DC/CIK cycles after chemotherapy had significantly prolonged median time-to-progression (7.3 vs. 6.2 months; *p*=0.034) and higher 3-year overall survival (23.3% vs. 6.7%; *p*=0.037) (56). However, side effects such as skin reactions and non-infectious fevers remain considerations in clinical management (56). Similarly, in stage IIIB patients, combining DC/CIK therapy with chemoradiotherapy improved objective response rate (83.3% vs. 54.5%) and 12-month survival (63.3% vs. 42.4%), along with superior T cell subset recovery and performance status (57). More recently, a phase Ib trial investigated the combination of CIK cells, platinum-based chemotherapy, and the anti-PD-1 antibody sintilimab in treatment-naïve stage IIIB-IV NSCLC (NCT03987867) (58). The regimen achieved an objective response rate of 82.4% and a disease control rate of 100%, with median PFS of 19.3 months (58). However, treatment-related adverse events occurred in 94.1% of patients, including one grade 5 immune-related pneumonia, underscoring the need to balance efficacy with safety in intensified regimens (58). Together, these studies support the therapeutic potential of DC/CIK therapy across various NSCLC stages, especially when used in multimodal strategies. Nonetheless, the lack of consistent stage-specific analyses across trials limits precise interpretation. Future studies should incorporate stratified survival outcomes based on TNM staging and immune phenotypes to guide individualized treatment optimization.

### 4.3 Barrier and promise of DC/CIK cell therapy

Despite its clinical potential, DC/CIK cell therapy faces significant barriers, primarily due to the immunosuppressive TME in advanced NSCLC. This environment is dominated by immunosuppressive cell populations such as MDSCs, Tregs, and M2-polarized tumor-associated macrophages (TAMs) (59). These cells secrete inhibitory cytokines, including TGF-β and vascular endothelial growth factor (VEGF), and recruit pro-tumorigenic immune subsets that undermine the efficacy of DC-based vaccines by impairing CD8^+^ T-cell activation (60). Attempts to modulate the TME have included strategies such as cyclophosphamide preconditioning to deplete Tregs or all-trans retinoic acid (ATRA) to induce MDSC differentiation (61, 62). However, these approaches can exhibit dual effects. For example, ATRA may paradoxically stabilize Tregs under inflammatory conditions, thereby attenuating therapeutic outcomes (63). In addition, tumor-derived inflammatory mediators such as VEGF and IL-6 exacerbate immune dysfunction by promoting MDSC infiltration and skewing T-helper responses toward a Th2 phenotype (60). Moreover, suboptimal antigen selection, such as the use of nonspecific tumor lysates or limited antigen cocktails, may result in either inadequate immunogenicity or off-target autoimmunity (64, 65).

A major limitation of DC/CIK therapy is the lack of validated predictive biomarkers to identify patients most likely to benefit. Unlike checkpoint blockade or TIL therapy, where PD-L1 expression or neoantigen load may offer guidance, reliable biomarkers for DC/CIK efficacy remain undefined. Candidate indicators such as baseline immune cell composition (e.g., CD8^+^/Treg ratio), cytokine profiles, or TME-related gene signatures are under investigation, but none have reached clinical implementation. Encouragingly, emerging strategies may enhance both efficacy and patient stratification. Reprogramming TAMs toward an M1 phenotype, blocking immunosuppressive cytokines with VEGF inhibitors, and engineering DCs to present tumor-specific or neoantigen-derived peptides represent promising approaches to enhance TME remodeling and therapeutic precision (66). Co-administration of DC vaccines with immune checkpoint inhibitors, such as PD-1 blockade, may further circumvent immune suppression while generating durable antitumor memory, an advantage that surpasses the transient efficacy of antibody monotherapies (66). Future directions should focus on refining antigen selection by prioritizing highly immunogenic, tumor-exclusive targets and validating optimized peptide cocktails in large-scale clinical trials. Simultaneously, advancements in delivery protocols and manufacturing technologies will be essential to improve scalability and consistency. By integrating TME-modulating agents, precision antigen loading, and immune checkpoint modulation, DC/CIK therapy has the potential to evolve into a robust and durable immunotherapeutic strategy for patients with refractory NSCLC.

## 5 CAR-T cell therapy

### 5.1 Mechanism of CAR-T cell therapy

**Figure 2:**
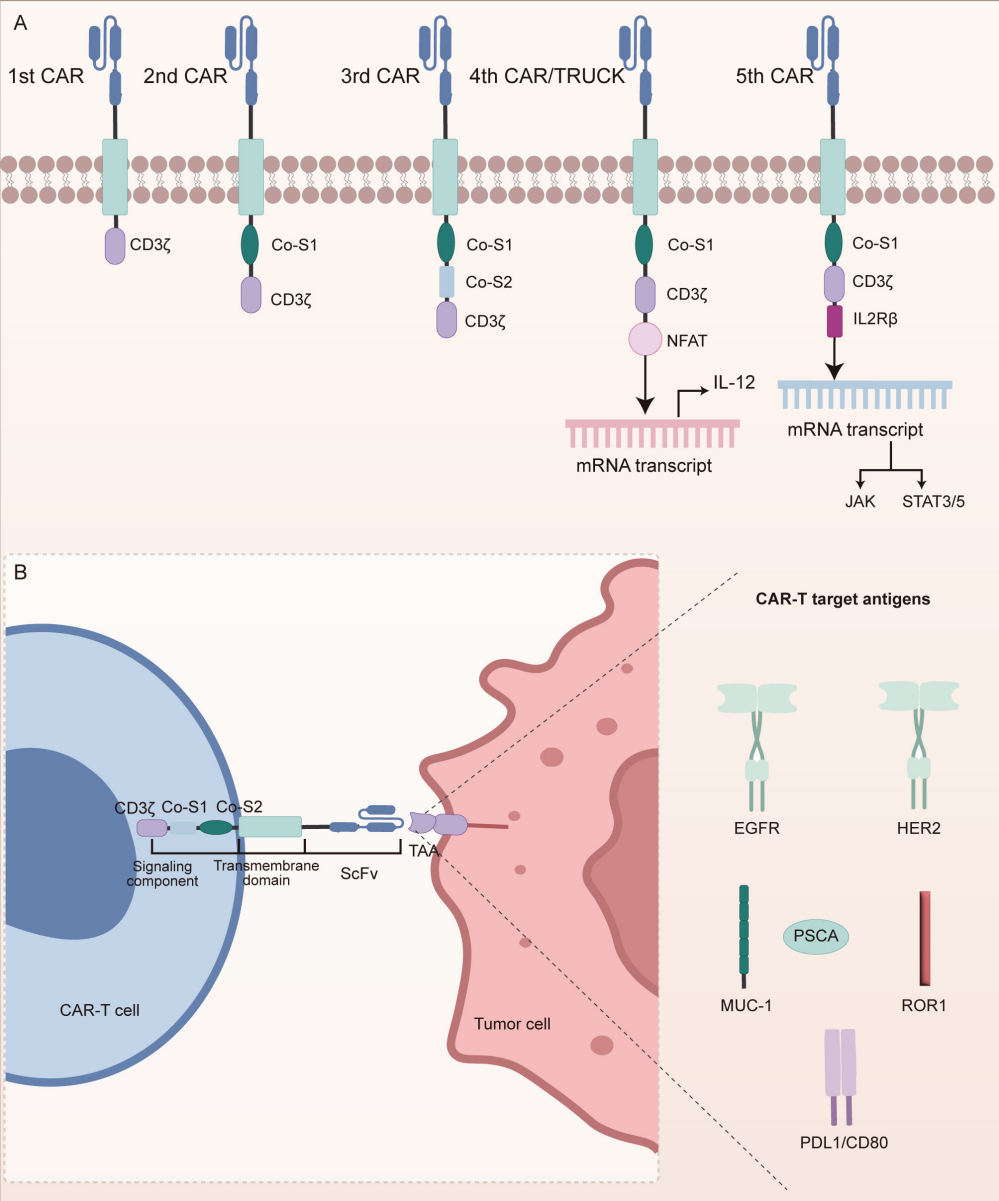
Structural evolution of CAR constructs and antigen targets in NSCLC. **(A)** Progressive generations of CARs have been structurally optimized to improve T cell activation, persistence, and anti-tumor efficacy. First-generation CARs contain only the CD3ζ signaling domain. Second-generation CARs incorporate a single co-stimulatory domain to enhance T cell survival. Third-generation CARs integrate two co-stimulatory domains for stronger activation. Fourth-generation CARs introduce inducible cytokine secretion to modulate the tumor microenvironment. Fifth-generation CARs add IL-2Rβ to activate JAK-STAT signaling pathways, further enhancing T cell proliferation and function. **(B)** CAR-T cells in NSCLC primarily target tumor-associated antigens (TAAs), including EGFR, HER2, MUC1, PSCA, ROR1, PD-L1, and CD80. These antigen-specific CAR-T cells activate cytotoxic signaling cascades through CD3ζ and co-stimulatory modules upon antigen recognition.

The therapeutic core of chimeric antigen receptor T (CAR-T) cell therapy lies in the reengineering of T cell antigen recognition and activation pathways through synthetic biology techniques (**Figure 1D**) (67). CAR molecules are composed of a single-chain variable fragment (scFv) that specifically binds TAAs, a transmembrane domain, and intracellular signaling components such as CD3ζ and co-stimulatory domains (e.g., CD28 or 4-1BB) (67). The structural refinement of CAR designs has progressively enhanced T cell function (**Figure 2A**). The extracellular spacer/hinge region optimizes antigen accessibility and signal transduction, while intracellular modifications regulate activation and persistence (68). First-generation CARs contained only the CD3ζ domain to initiate T cell activation (68). Second-generation CARs incorporated a single co-stimulatory domain, such as CD28 or 4-1BB, to enhance T cell persistence (68). Third-generation CARs further integrated two co-stimulatory domains to improve signaling potency (68). Fourth-generation CARs introduced inducible cytokine secretion to modulate the TME (68). Fifth-generation CARs, derived from the second generation, incorporated IL-2Rβ to activate the JAK-STAT3/5 pathway, reinforcing T cell proliferation and function (68). Engineered T cells are genetically modified via viral vectors or non-viral transposon systems and expanded ex vivo with IL-2/IL-15 (69). Upon reinfusion, CAR-T cells recognize tumor surface antigens via their scFv domains, form immunological synapses, and release perforin and granzyme B to mediate direct cytotoxicity. They also secrete pro-inflammatory cytokines such as IFN-γ and TNF-α, which recruit innate immune components to amplify the anti-tumor response (69).

In NSCLC, CAR-T cell therapy primarily targets TAAs, as illustrated in **Figure 2B**. Epidermal growth factor receptor (EGFR), a member of the ErbB receptor tyrosine kinase family, contains tumor-specific epitopes within its extracellular domain and is mutated or overexpressed in over 60% of NSCLC cases, promoting tumor proliferation and metastasis (70). EGFR-targeted CAR-T cells recognize these epitopes to induce direct tumor cell lysis (70). Similarly, transmembrane glycoproteins such as mucin 1 (MUC1) and prostate stem cell antigen (PSCA) are aberrantly overexpressed in NSCLC (71). PSCA-targeted CAR-T cells have demonstrated the capacity to delay tumor progression, whereas MUC1, particularly its glycosylated tumor-specific isoform (e.g., TnMUC1), offers a highly specific target for immunotherapy (71). Human epidermal growth factor receptor 2 (HER2), another ErbB family receptor, plays an oncogenic role in NSCLC through aberrant activation (72, 73). CAR-T cells directed against HER2 recognize its extracellular domain to initiate cytotoxic responses. In addition, immune checkpoint molecules such as PD-L1 and CD80, which suppress T cell function, are also therapeutic targets (74, 75). Dual-targeting CAR-T constructs against PD-L1 and CD80 not only block inhibitory signals but simultaneously activate co-stimulatory pathways, thereby reshaping the immunosuppressive TME (74, 75). Receptor tyrosine kinase-like orphan receptor 1 (ROR1), selectively expressed in NSCLC, possesses a unique extracellular structure that facilitates high-specificity CAR recognition (76). All these targeting strategies rely on the surface expression of CAR-encoded scFvs to bind tumor antigens with high specificity. Upon antigen engagement, intracellular signaling modules such as CD3ζ and 4-1BB are activated, initiating a cascade of cytotoxic responses. Moreover, recent engineering innovations, such as dual-antigen targeting and glycoform-specific recognition, are being employed to enhance tumor selectivity and overcome resistance mechanisms. These advances have laid the foundation for early-phase clinical trials in NSCLC, where the safety and feasibility of CAR-T therapy are now being actively explored.

### 5.2 Clinical trials of CAR-T cell therapy

Recently, many clinical trials on CAR-T cell therapy have made major progress (**Table 4**). EGFR-targeted CAR-T therapy has demonstrated variable clinical outcomes in advanced NSCLC, with efficacy influenced by CAR design, gene transfer method, and dosing strategies. In a phase I/II trial (NCT03182816), Zhang et al. utilized a non-viral *piggyBac* transposon system to generate EGFR-CAR-T cells, thus circumventing risks associated with viral vector-mediated genomic integration (77). Among 9 patients with refractory EGFR-positive NSCLC, CAR-T cell persistence was observed in 89% (8/9) of cases (77). One patient achieved a partial response lasting more than 13 months, while six experienced stable disease. The reported median PFS and OS were 7.13 and 15.63 months, respectively (77). Despite improved safety, efficacy remained modest. This may be attributed to EGFR’s low-level expression in normal tissues, which constrains dosing due to on-target/off-tumor risks, and to the immunosuppressive TME, including PD-L1 expression and T-cell exhaustion, which limit CAR-T expansion and activity. Lack of tumor biopsies further precluded evaluation of intratumoral response. Future strategies, such as combining with PD-1/PD-L1 blockade or engineering CAR-Ts to resist exhaustion, may improve outcomes. Subsequent trials have explored refinements. In NCT01869166, EGFR-CAR-T cells administered to biomarker-selected patients (≥50% EGFR expression) showed no severe adverse effects, supporting autologous safety. Another trial (NCT04153799) using CXCR5-modified EGFR-CAR-Ts achieved two partial responses and five long-lasting stable diseases among 11 patients, suggesting enhanced tumor trafficking. These findings underscore the need for multi-dimensional optimization (1): enhancing tumor selectivity to minimize off-tumor effects; (2) improving persistence through cytokine support or memory-enriched phenotypes; and (3) integrating CAR-T with checkpoint inhibitors to overcome adaptive resistance in solid tumors.

**Table 4:** Summary of key clinical trials on CAR-T cell therapy.

Clinical trials	Target	Phase	Design features	Key outcomes	Challenges/Limitations
NCT03182816	EGFR	I/II (2017)	EGFR-CAR-T cells are generated using a non-viral piggyBac transposon system to avoid genomic integration risks	-89% CAR-T persistence (8/9 patients); -1 partial response (>13 months); -Median PFS: 7.13 months; OS: 15.63 months.	Limited efficacy, possibly due to off-target toxicity from EGFR expression in normal tissues
NCT01869166	EGFR	I/II (2015)	Autologous peripheral blood-derived EGFR-CAR-T cells targeting patients with high EGFR expression (≥50%)	-No severe toxicity events reported.	Efficacy data not clearly reported; further validation needed in biomarker-selected populations
NCT04153799	EGFR (CXCR5-modified)	I (2020)	EGFR-CAR-T cells engineered with CXCR5 chemokine receptor to enhance tumor homing	-2 partial responses -5 stable diseases (up to 8 months).	Heterogeneous dosing may influence outcomes; dosing optimization required
NCT03198052	PSCA & MUC1	I (2020)	Third-generation PSCA-CAR-T combined with MUC1 targeting strategy	-PSCA-CAR-T delayed tumor progression; -MUC1-CAR-T ineffective in PDX models.	Synergistic effects of dual targeting require further validation; limitations of MUC1 targeting remain unresolved
NCT02713984	HER2	I/II (2020)	HER2-CAR-T (4-1BB/CD3ζ signaling domain)	-Trial terminated due to off-tumor toxicity related to HER2 expression in normal tissues.	Severe toxicity from pan-cancer antigen targeting; need for improved tumor-specific strategies
NCT03060343 NCT03330834	PD-L1/CD80	I (2017/2020)	Dual-target CAR-T cells against PD-L1 and CD80 to block inhibitory signaling and activate costimulatory pathways	-Ongoing trials aiming to enhance CAR-T persistence and activity in the tumor microenvironment.	Effectiveness in overcoming TME suppression remains to be validated; complex regulatory signaling poses challenges
NCT02706392	ROR1	I (2021)	Autologous ROR1-CAR-T cells (engineered with iCasp9M28z)	-Successful peripheral expansion but insufficient tumor infiltration; No clinical responses.	Rapid CAR-T exhaustion and antigen heterogeneity limit durability and efficacy

CAR-T, Chimeric Antigen Receptor T Cell; EGFR, Epidermal Growth Factor Receptor; CXCR, C-X-C Motif Chemokine Receptor 5; PSCA, Prostate Stem Cell Antigen; MUC1, Mucin 1; PDX, Patient-Derived Xenograft; HER2, Human Epidermal Growth Factor Receptor 2; PD-L1, Programmed Death Ligand 1; TME, Tumor Microenvironment; ROR1, Receptor Tyrosine Kinase-like Orphan Receptor 1; OS, Overall Survival; PFS, Progression-Free Survival.

Despite the promise of EGFR-targeted CAR-T cells, identifying suitable alternative targets remains challenging. Although antigens such as MUC1 and PSCA are overexpressed in NSCLC, CAR-T cells targeting MUC1 failed to inhibit tumor growth in patient-derived xenograft models. Conversely, third-generation CAR-T cells directed against PSCA showed delayed tumor progression in a phase I study (NCT03198052), highlighting the potential benefit of combinatorial antigen targeting strategies. HER2-targeted CAR-T therapy, however, was hampered by on-target/off-tumor toxicity. Despite HER2’s oncogenic role, protocol modifications were required due to safety concerns, ultimately leading to the discontinuation of a phase I/II trial (NCT02713984). This case underscores the inherent risk of targeting pan-carcinoma antigens that are also expressed in normal tissues. In addition to antigen selection, the immunosuppressive TME poses a major barrier to CAR-T cell efficacy in NSCLC. Molecules such as PD-L1, CD80, and CD86 inhibit CAR-T cell function and limit their persistence. To address this, ongoing phase I trials (NCT03060343, NCT03330834) are investigating CAR-T constructs targeting PD-L1 and CD80, aiming to simultaneously interrupt checkpoint signaling and activate co-stimulatory pathways, thereby improving CAR-T cell survival and functionality within the TME. The complexity of the TME is further highlighted by the phase I trial of ROR1-directed CAR-T cells (NCT02706392). Despite robust peripheral expansion following lymphodepletion, the CAR-T cells failed to adequately infiltrate tumor sites and rapidly became exhausted, ultimately leading to no observable clinical responses (78). Furthermore, antigen heterogeneity remains a formidable obstacle. Tumor cells may escape immune surveillance by downregulating the targeted antigen. Although preclinical models demonstrated robust efficacy of ROR1-directed CAR-T cells in eliminating NSCLC organoids, clinical translation was limited by insufficient persistence and heterogeneous antigen expression (78). These challenges underscore the urgent need for adaptive CAR-T cell designs capable of responding to dynamic antigen landscapes and minimizing the risk of immune escape and tumor relapse.

### 5.3 Barrier and promise of CAR-T cell therapy

Despite the transformative success of CAR-T cell therapy in hematologic malignancies, its application in NSCLC remains limited by numerous challenges. One of the foremost obstacles is on-target/off-tumor toxicity, wherein CAR-T cells inadvertently attack healthy tissues that express TAAs shared with malignant cells, potentially resulting in life-threatening multi-organ damage (79). In contrast to hematologic cancers, where CAR-T therapy has achieved durable remissions, more than half of treated patients still relapse, and outcomes in solid tumors remain modest. These setbacks stem from tumor antigen heterogeneity, poor CAR-T infiltration, and limited persistence, underscoring the need for strategies that prolong antitumor responses while minimizing off-tumor toxicity (80). Strategies to mitigate this risk include targeting more tumor-specific epitopes, such as EGFR variant III, or engineering low-affinity CARs that preferentially bind to tumor cells exhibiting high antigen density (81, 82). Neurological toxicities, including seizures and confusion, have also been reported but remain mechanistically elusive (79). These are currently managed with corticosteroids, emphasizing the urgent need for deeper mechanistic understanding and the development of neurotoxicity-specific predictive biomarkers, such as serum neurofilament light chain or early cytokine signatures (79). In addition, CRS, a systemic inflammatory response driven by elevated levels of cytokines such as IL-6 and TNF-α, poses a major safety concern (83). Clinical management of CRS involves timely administration of IL-6 receptor antagonists (e.g., tocilizumab) or TNF-α inhibitors (e.g., infliximab), along with continuous monitoring of inflammatory markers (84). Emerging biomarkers such as baseline IFN-γ or ferritin levels have shown promise for early CRS prediction, but remain to be prospectively validated in NSCLC.

A fundamental limitation in NSCLC is the scarcity of truly tumor-specific antigens within its heterogeneous tumor landscape. Efforts to overcome this include pharmacological upregulation of target antigens using agents such as all-trans-retinoic acid, and engineering CAR-T cells capable of recognizing antigens expressed at low densities (85, 86). However, the absence of validated biomarkers to predict antigen density, distribution, and CAR-T engagement limits rational target selection and patient stratification. Moreover, the immunosuppressive TME, characterized by hypoxia, metabolic deprivation, and immunoinhibitory cytokines, profoundly impairs CAR-T cell function and persistence (87). Combinatorial therapeutic strategies are therefore being explored, such as co-administration of immune checkpoint inhibitors and the design of “armored” CAR-T cells that secrete pro-inflammatory cytokines (e.g., IL-12, IL-7/IL-15) to recondition the TME and restore immune effector function (88). Another significant barrier is poor CAR-T cell infiltration into tumor tissues, exacerbated by abnormal tumor vasculature and mismatches between tumor-secreted chemokines and T cell-expressed chemokine receptors (89, 90). To enhance homing, CAR-T cells are being engineered to express specific chemokine receptors, such as CXCR2 or CCR4, that correspond to the chemokine profiles of NSCLC tumors (89, 90). Furthermore, antigen escape, wherein tumor cells downregulate or lose the targeted antigen, remains a persistent cause of therapeutic resistance (91). To address this, multi-specific CAR designs have been developed to target multiple TAAs simultaneously, thereby reducing the likelihood of immune evasion and disease relapse (91). Despite these considerable barriers, ongoing innovations in CAR engineering, including switchable CARs, universal platforms, and armored CAR-T constructs, demonstrate the therapy’s remarkable adaptability (92). By integrating refined antigen selection, strategic modulation of the tumor microenvironment, and advanced precision-targeting modalities, CAR-T cell therapy holds substantial promise for overcoming the unique complexities of NSCLC and achieving durable clinical responses.

## 6 Comparison of ACT modalities and safety management

To provide a comprehensive perspective on ACTs for NSCLC, we conducted a comparative evaluation of four representative modalities, as shown in **Table 5**. These platforms differ significantly in their efficacy, safety, scalability, and clinical feasibility. While potency is a key determinant of therapeutic value, real-world application also depends heavily on safety profiles and the ability to manage toxicity. In this context, treatment selection must balance antitumor efficacy with tolerability, particularly in the setting of advanced NSCLC, where patient fragility and tumor burden amplify the risk of treatment-related adverse events. Safety profiles across these modalities vary considerably.

**Table 5:** Comparative overview of adoptive cell therapies in NSCLC.

Parameter	LAK	DC/CIK	TIL	CAR-T
Efficacy	Low; limited tumor infiltration and cytotoxicity	Modest as monotherapy; improved with chemotherapy or ICIs	Promising in ICI-refractory tumors; under active investigation	Moderate in NSCLC (higher in hematologic malignancies)
Tumor Specificity	Non-specific cytotoxicity	Partial specificity via DC antigen presentation	Broad neoantigen recognition via endogenous TCRs	Engineered for specific TAAs; risk of off-tumor effects
Major Toxicities	Severe (e.g., high-dose IL-2 toxicity, vascular leak syndrome)	Mild (e.g., fever, rash); rarely severe	Toxicities mainly from LD and IL-2 (e.g., cytopenia, cardiomyopathy)	CRS, neurotoxicity, on-target/off-tumor effects
Safety Profile	Poor; high incidence of grade≥3 AEs	Favorable; well tolerated in most patients	Moderate; safety improved with shorter LD or lower IL-2 dosing	Moderate to poor; requires intensive monitoring
Manageability	Difficult; dose-limiting IL-2 toxicities	Generally outpatient-compatible; minimal supportive care needed	Ongoing IL-2 optimization; engineered IL-2 in early-phase trials	IL-6 inhibitors (e.g., tocilizumab), corticosteroids used
Manufacturing Complexity	Simple expansion of PBMCs; no tumor specificity required	Low to moderate (short culture cycle, no gene editing)	Moderate (requires surgical sample and ex vivo expansion)	High (viral transduction, GMP-grade production)
Scalability	Scalable, but obsolete due to low efficacy and high toxicity	More scalable in clinical settings	Challenging; improving with automation and centralization	Limited; patient-specific and technically demanding
Cost	Low production cost, but high due to hospitalization	Relatively low	Moderate to high (personalized product)	High
Development Status	Obsolete due to poor clinical benefit and safety	Widely used in Asia; limited randomized data elsewhere	Early-phase NSCLC trials show potential	Phase I-II trials ongoing

LAK, Lymphokine-Activated Killer; DC/CIK, Dendritic Cell–Cytokine-Induced Killer; TIL, Tumor-Infiltrating Lymphocyte; CAR-T, Chimeric Antigen Receptor T Cell; NSCLC, Non-Small Cell Lung Cancer; ICI, Immune Checkpoint Inhibitor; TAA, Tumor-Associated Antigen; TCR, T Cell Receptor; IL-2, Interleukin-2; LD, Lymphodepletion; CRS, Cytokine Release Syndrome; AE, Adverse Event; PBMC, Peripheral Blood Mononuclear Cell; GMP, Good Manufacturing Practice.

LAK therapy, once widely explored, has largely been abandoned due to poor tumor specificity and unacceptable toxicities related to high-dose IL-2 administration, such as vascular leak syndrome and systemic inflammation. In contrast, DC/CIK therapy offers a favorable safety record, with adverse events generally limited to mild, self-limiting symptoms such as low-grade fever, rash, or fatigue (55, 56, 93). Importantly, when used in combination with chemotherapy, DC/CIK therapy may attenuate chemotherapy-related toxicities, although its clinical efficacy remains modest (53). CAR-T therapy demonstrates strong tumor-killing capacity but is accompanied by well-documented high-grade toxicities, including CRS, neurotoxicity, and on-target/off-tumor effects, which often necessitate inpatient monitoring and immunosuppressive intervention (79, 81, 82). For example, the use of immunosuppressive agents such as corticosteroids and IL-6 inhibitors (94). However, the precise mechanisms of neurotoxicity, including symptoms such as confusion, aphasia, and seizures, remain unclear. Moreover, long-term effects such as persistent cytopenia, B-cell aplasia, and the potential for clonal expansion or insertional mutagenesis require ongoing surveillance, particularly with the use of viral vectors (79, 81, 82). TIL therapy, while autologous and relatively tumor-specific, is complicated by toxicities arising from lymphodepleting chemotherapy and high-dose IL-2. Recent studies suggest that toxicity may be mitigated through shorter LD regimens and engineered IL-2 analogs, potentially improving its safety-efficacy balance (95). Genetic modifications, such as PDCD1 or CISH knockout and CXCR2 overexpression, have shown potential to enhance TIL function, persistence, and tumor homing (96, 97). These strategies may allow for reduced IL-2 dosing or lymphodepletion intensity, improving safety. However, they also increase manufacturing complexity and deviate from the minimally manipulated nature of traditional TILs, raising new regulatory and logistical challenges. Taken together, these profiles suggest that future optimization of ACTs must consider not only therapeutic strength but also the clinical manageability of adverse events, ideally through biomarker-informed patient selection and dose-adaptive strategies.

## 7 Summary and prospects

Adoptive cell therapies have emerged as promising strategies in NSCLC, with each platform offering distinct mechanisms and clinical characteristics. While LAK and DC/CIK therapies laid the groundwork for cellular immunotherapy, their limited specificity and modest efficacy have constrained further development. In contrast, TIL and CAR-T therapies represent the current forefront: the former capitalizing on natural neoantigen recognition, and the latter on synthetic precision targeting. Their continued refinement reflects the growing emphasis on tailoring cellular interventions to tumor biology.

However, a major barrier to the success of adoptive cell therapies in NSCLC is the considerable molecular and immunological heterogeneity of the disease. Distinct oncogenic mutations correlate with differing immune landscapes. For instance, EGFR-mutant tumors are frequently characterized by low TMB, reduced neoantigen load, minimal CD8^+^ T-cell infiltration, and downregulated MHC expression, contributing to an immunologically “cold” TME and poor response to both checkpoint inhibitors and ACTs (98). In contrast, KRAS-mutant tumors, particularly those co-mutated with TP53, tend to display higher TMB, increased chemokine-driven T-cell recruitment, and an inflamed immune phenotype, making them more responsive to TIL or CAR-T therapies (99). However, subsets such as KRAS/STK11 co-mutated tumors are often immunologically inert despite high TMB, suggesting that TMB alone is not a sufficient predictor of response (99, 100). Additional modulators, including PD-L1 expression, antigen-presenting machinery defects, and suppressive stromal or myeloid components, further influence therapeutic efficacy (100). Moving forward, biomarker-driven patient stratification based on integrated genomic and immunologic profiling will be essential to guide ACT selection and improve clinical outcomes (101).

Looking ahead, overcoming the limitations of each ACT modality will require a multifaceted approach. For TIL therapy, strategies such as genetic enhancement (e.g., PD-1, CISH knockout, IL-7 transduction) aim to improve persistence and function while preserving its autologous nature. For CAR-T therapy, innovations such as dual-targeting, switchable receptors, and safety switches are addressing key barriers including antigen escape and off-tumor toxicity. Given their complementary strengths, TIL’s adaptability to heterogeneity and CAR-T’s potency against defined targets, hybrid approaches or rational combinations with ICIs, vaccines, or oncolytic viruses may offer synergistic benefits. Meanwhile, LAK and DC/CIK therapies, though more limited in current scope, may still find value within combination regimens or as lower-toxicity options in select patients. Ultimately, personalized design based on tumor-specific immune contexture will be essential to fully realize the potential of ACT in NSCLC.
